# Supplementary material for: 4-Arylthiosemicarbazide Derivatives as Toxoplasmic Aromatic Amino Acid Hydroxylase Inhibitors and Anti-inflammatory Agents
Source: Int J Mol Sci. 2022 Mar 16;23(6):3213. doi: 10.3390/ijms23063213 (PMC8955734; doi:10.3390/ijms23063213)
Supplement: Supplementary file 1 [file ijms-23-03213-s001.zip › ijms-1631473-supplementary.pdf]

## Supplementary Material

# 4-Arylthiosemicarbazide derivatives as toxoplasmic aromatic amino acid hydroxylase inhibitors and anti-inflammatory agents

**Adrian Bekier**[1], **Anna Brzostek**[2], **Agata Paneth**[3], **Bożena Dziadek**[1], **Jarosław Dziadek**[2] , **Justyna Gatkowska**[1] and **Katarzyna Dzitko**[1]

[1] University of Lodz, Faculty of Biology and Environmental Protection, Department of Molecular Microbiology, Banacha 12/16, 90-237 Lodz, Poland; adrian.bekier@biol.uni.lodz.pl (A.Be.); Bożena Dziadek (B.D.) bozena.dziadek@biol.uni.lodz.pl

[2] Laboratory of Genetics and Physiology of Mycobacterium, Institute of Medical Biology, Polish Academy of Sciences, 93-232 Lodz, Poland; abrzostek@cbm.pan.pl (A.Br.); jdziadek@cbm.pan.pl (J.D.)

[3] Medical University of Lublin, Faculty of Pharmacy, Department of Organic Chemistry, Chodzki 4a, 20-093 Lublin, Poland; agatapaneth@umlub.pl (A.P.)

## Table of contents:

Figure S1. SDS–PAGE of BSA from the control drug affinity responsive target stability assay.

Figure S2. Western blots of AAH1 from the drug affinity responsive target stability assay.

Table S1. The results of two-way ANOVA were conducted, and for significant comparisons, further analysis was performed using Dunnett's multiple comparisons test. The differences were considered significant with a p value < 0.05.

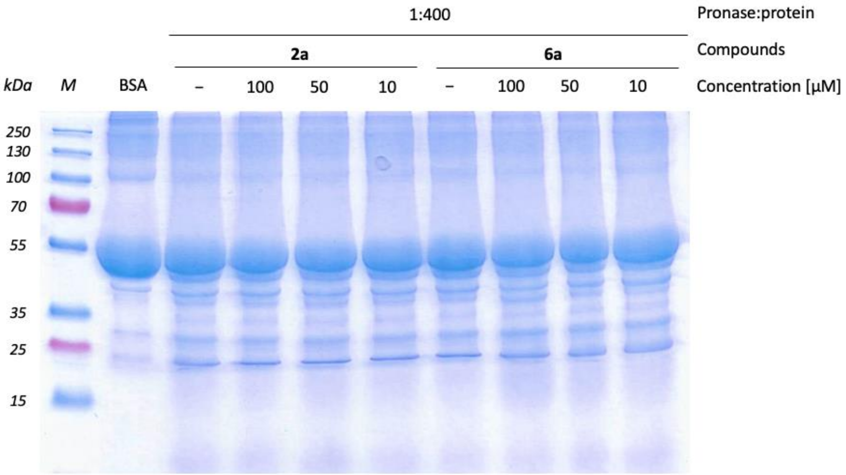


**Figure S1**. SDS–PAGE of BSA from the control drug affinity responsive target stability assay.

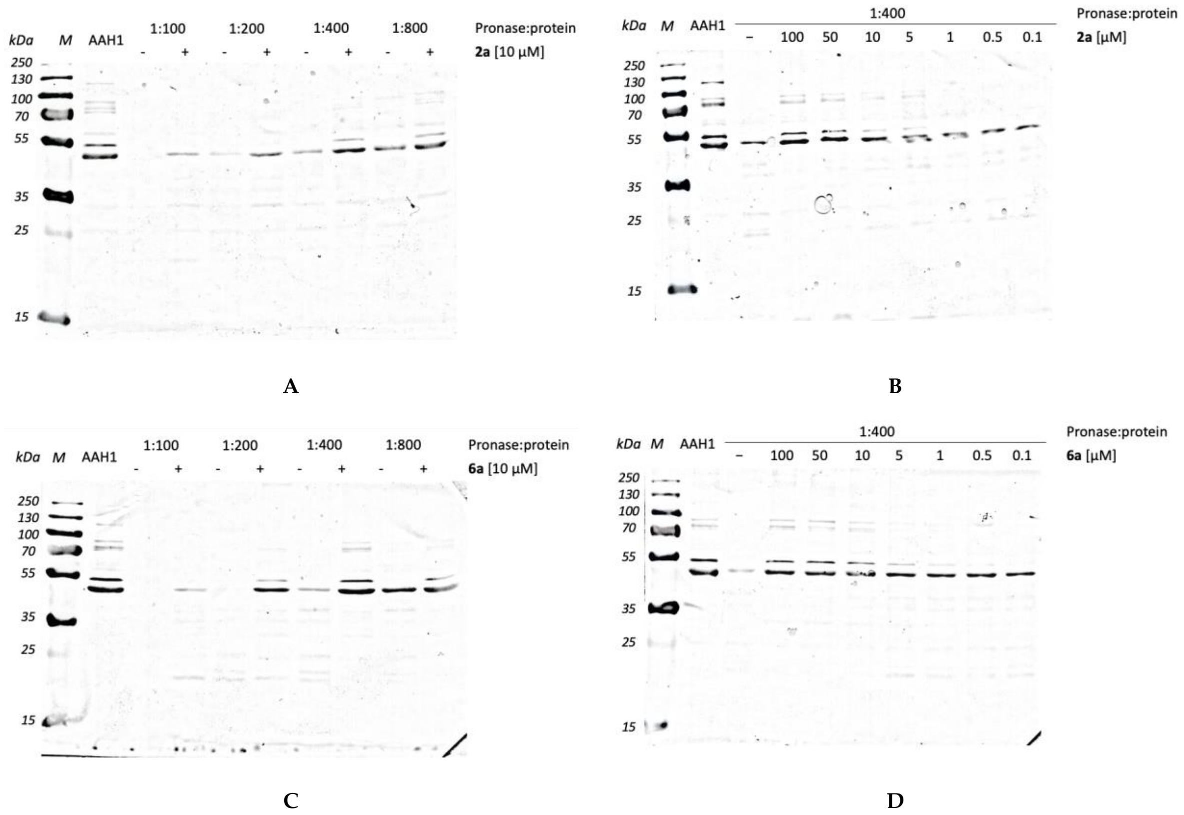


**Figure S2.** Western blots of AAH1 from the drug affinity responsive target stability assay. **A** Protection of AAH1 from proteolysis by **2a** in the presence of 10 μM of compound. **B.** Protection of AAH1 from proteolysis by **2a** in the presence of increasing concentrations of compound. **C.** Protection of AAH1 from proteolysis by **6a** in the presence of 10 μM of compound. **D.** Protection of AAH1 from proteolysis by **6a** in the presence of increasing concentrations of compound.

**Table S1** The results of two-way ANOVA were conducted, and for significant comparisons, further analysis was performed using Dunnett's multiple comparisons test. The differences were considered significant with a p value < 0.05.

| | **NF-κB induction in THP1-Blue™** | | | **INF-γ production** | | |
|---|---|---|---|---|---|---|
| | Predicted mean difference | Summary | P value | Predicted mean difference | Summary | P value |
| TLA | | | | | | |
| nt vs. 6a 2h a.s. | 1.079 | **** | <0.0001 | 13,06 | **** | <0,0001 |
| nt vs. 6a 2h b.s. | 1.079 | **** | <0.0001 | 12,26 | **** | <0,0001 |
| nt vs. 2a 2h a.s. | 0.5013 | **** | <0.0001 | 12,50 | **** | <0,0001 |
| nt vs. 2a 2h b.s. | 1.076 | **** | <0.0001 | 11,86 | **** | <0,0001 |
| BLA | | | | | | |
| nt vs. 6a 2h a.s. | 1.011 | **** | <0.0001 | 15,14 | **** | <0,0001 |
| nt vs. 6a 2h b.s. | 1.011 | **** | <0.0001 | 14,35 | **** | <0,0001 |
| nt vs. 2a 2h a.s. | 0.4389 | **** | <0.0001 | 14,59 | **** | <0,0001 |
| nt vs. 2a 2h b.s. | 1.003 | **** | <0.0001 | 14,75 | **** | <0,0001 |
| AAH1 | | | | | | |
| nt vs. 6a 2h a.s. | 1.657 | **** | <0.0001 | 21,04 | **** | <0,0001 |
| nt vs. 6a 2h b.s. | 1.914 | **** | <0.0001 | 19,92 | **** | <0,0001 |
| nt vs. 2a 2h a.s. | 0.8091 | **** | <0.0001 | 19,99 | **** | <0,0001 |
| nt vs. 2a 2h b.s. | 1.644 | **** | <0.0001 | 19,67 | **** | <0,0001 |
| AAH2 | | | | | | |
| nt vs. 6a 2h a.s. | 1.518 | **** | <0.0001 | 17,24 | **** | <0,0001 |
| nt vs. 6a 2h b.s. | 1.662 | **** | <0.0001 | 15,95 | **** | <0,0001 |
| nt vs. 2a 2h a.s. | 0.8798 | **** | <0.0001 | 16,92 | **** | <0,0001 |
| nt vs. 2a 2h b.s. | 1.466 | **** | <0.0001 | 16,20 | **** | <0,0001 |
| LPS | | | | | | |
| nt vs. 6a 2h a.s. | 1.661 | **** | <0.0001 | 41,17 | **** | <0,0001 |
| nt vs. 6a 2h b.s. | 1.809 | **** | <0.0001 | 40,69 | **** | <0,0001 |
| nt vs. 2a 2h a.s. | 0.7927 | **** | <0.0001 | 40,20 | **** | <0,0001 |
| nt vs. 2a 2h b.s. | 1.342 | **** | <0.0001 | 40,61 | **** | <0,0001 |
| not stimulated | | | | | | |
| nt vs. 6a 2h a.s. | 0.04591 | ns | 0.5062 | -0,7622 | ns | 0,4697 |
| nt vs. 6a 2h b.s. | 0.04576 | ns | 0.5090 | -1,063 | ns | 0,1917 |
| nt vs. 2a 2h a.s. | 0.04453 | ns | 0.5320 | -0,4559 | ns | 0,8366 |
| nt vs. 2a 2h b.s. | 0.04428 | ns | 0.5367 | -0,6224 | ns | 0,6416 |
